# Immune-Related Gene Expression Patterns in GPV- or H9N2-Infected Goose Spleens

**DOI:** 10.3390/ijms17121990

**Published:** 2016-12-01

**Authors:** Shun Chen, Anqi Wang, Lipei Sun, Fei Liu, Mingshu Wang, Renyong Jia, Dekang Zhu, Mafeng Liu, Qiao Yang, Ying Wu, Kunfeng Sun, Xiaoyue Chen, Anchun Cheng

**Affiliations:** 1 Institute of Preventive Veterinary Medicine, Sichuan Agricultural University, Chengdu 611130, China; anqiwang77@163.com (A.W.); 13408051770@163.com (L.S.); mshwang@163.com (M.W.); cqrc_jry@163.com (R.J.); liumafengra@163.com (M.L.); yangqiao721521@sina.com (Q.Y.); yingzi_no1@126.com (Y.W.); sunkunfeng1981@163.com (K.S.); 2 Avian Disease Research Center, College of Veterinary Medicine of Sichuan Agricultural University, Chengdu 611130, China; zdk24@163.com (D.Z.); sophia_cs@yeah.net (X.C.); 3 Key Laboratory of Animal Disease and Human Health of Sichuan Province, Sichuan Agricultural University, Chengdu 611130, China; liufei@sicau.edu.cn

**Keywords:** identical expression profile, systemic transcriptome, GPV, H9N2

## Abstract

Goose parvovirus (GPV) and avian influenza virus subtype H9N2 are single-stranded DNA (ssDNA) and single-stranded RNA (ssRNA) viruses, respectively, both of which can spread in goslings and cause a significant economic loss. To explore the comprehensive transcriptome of GPV- or H9N2-infected goose spleens and to understand the immune responses induced by a DNA virus (GPV) or a RNA virus (H9N2), RNA-seq was performed on the spleens of goslings at the fifth day post infection. In the present study, 2604 and 2409 differentially expressed unigenes were identified in the GPV- and H9N2-infected groups, respectively. Through KEGG pathway enrichment analyses, the up-regulated transcripts in the two virus-infected groups were mainly involved in immune-related pathways. In addition, the two virus-infected groups displayed similar expression patterns in the immune response pathways, including pattern-recognition receptor signaling pathways, the antigen processing and presentation pathway, the NF-κB signaling pathway and the JAK-STAT signaling pathway, as well as cytokines. Furthermore, most of the immune-related genes, particularly TLR7, TRAF3, Mx, TRIM25, CD4, and CD8α, increased in response to GPV and H9N2 infection. However, the depression of NF-κB signaling may be a mechanism by which the viruses evade the host immune system or a strategy to achieve immune homeostasis.

## 1. Introduction

Over the past years, the H5N1 influenza virus, originating from geese (*Anser cygnoides*), has been regarded as the proposed contributor to the human influenza-like illness that broke out in Hong Kong in 1997 [1] and continued to circulate in geese in Southeastern China, indicating that the goose is essential in the zoonotic transmission of the H5N1 influenza virus and is a potential transmitter of influenza viruses [2]. Moreover, the goose is an important transmitter and natural reservoir for many avian viruses, such as goose parvovirus (GPV) and the H9N2 influenza virus (H9N2).

GPV is the etiological agent of Derzsy’s disease [3], which can cause high morbidity and mortality in goslings and Muscovy ducklings [4]. Moreover, it was reported recently that GPV might be a contributor to the novel GPV (N-GPV), which causes beak atrophy and dwarfism syndrome (BADS) of commercial Cherry Valley duck flocks, with a morbidity rate between 10% and 30%, and even up to 50% in Northern China, since March 2015 [5]. The H9N2 influenza virus is currently widespread in poultry, which can transmit to humans and cause influenza [6]. Although H9N2 leads to mild morbidity, the co-circulation of H9N2 with H5N1 may increase the possibility of genetic reassortment and pose the threat of a pandemic [7]. Considering the harmfulness of these two viruses, many studies concerning the virology of GPV and H9N2 have been performed. However, the molecular mechanisms of the host-pathogen interaction have not yet been fully described. In addition, GPV and H9N2 are a small single-stranded DNA (ssDNA) virus and a single-stranded RNA (ssRNA) virus, respectively. Although the IFN-mediated immune response of the host cell during viral infections was partially described [8], much less is known about the antiviral response of the goose against infection at the molecular level, as well as the effects of infection with different pathogens on the expression patterns of host genes.

With the rapid development of high-throughput RNA sequencing (RNA-seq) technology, this success has the potential to explore the comprehensive transcriptional landscape in the host upon virus infection [9,10]. In this study, RNA-seq performed on the spleen tissues of GPV- or H9N2-infected geese was used to investigate the systemic alteration of the host gene expression after infections and to reveal the interaction mechanisms between the host and viruses. Moreover, our data demonstrated the subtle transcriptional landscape of goose spleen tissues infected by GPV and H9N2, which may provide valuable information for the immune response to diverse nucleic acid viruses in the goose spleen.

## 2. Results

### 2.1. Global Characteristics in Response to GPV and H9N2

**Figure 1:**
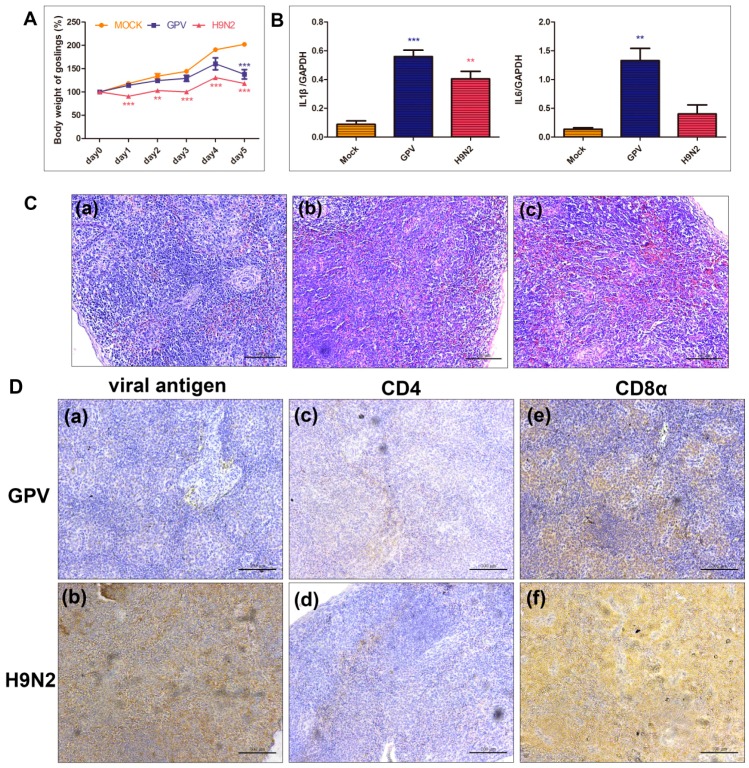
Global characteristics of goslings after infection with GPV and H9N2. (**A**) Body weight change of GPV- and H9N2-infected goslings from day 0 to 5. ** *p* ≤ 0.01, *** *p* ≤ 0.001; (**B**) the expression level of IL-1β and IL-6 in the spleen tissues of GPV- and H9N2-infected goslings. ** *p* ≤ 0.01, *** *p* ≤ 0.001; (**C**) histological changes in goose spleens infected with GPV and H9N2 at 5 dpi. The spleen tissues from (**a**) mock-, (**b**) GPV-, and (**c**) H9N2-infected goslings were stained with H &E, and diffuse hemorrhage was observed in either of virus-infected spleen sections, scale bar = 100 µm; and (**D**) Detection of (**a**) GPV and (**b**) H9N2 antigen, as well as (**c**,**d**) CD4- and (**e**,**f**) CD8α-positive cells by immunohistochemical analysis. The dark brown represents positive signs for viral antigen, CD4, or CD8α molecules, scale bar = 100 µm.

To investigate the effects of the two viruses on goslings, the body weights and histopathological changes in the spleen of the GPV- or H9N2-infected goslings were determined. The increase in the body weights of the infected goslings was inhibited. As shown in Figure 1A, the body weights of the mock-infected goslings increased steadily. However, the H9N2-infected goslings developed significantly slower than mock-infected goslings. Although the body weight of the goslings infected with GPV maintained the increase from one day post infection (dpi) to 4 dpi, a significant loss of body weight was observed at 5 dpi when the GPV-infected goslings were extremely significantly slim compared with mock-infected goslings. Moreover, GPV and H9N2 elicited higher expression of IL-1β and IL-6 than the mock-infected group, indicating that the host initiated an immune response to virus invasion (Figure 1B). In addition, diffuse hemorrhage was observed in the spleen samples from either of virus-infected goslings (Figure 1C). Meanwhile, the H9N2 antigen was distributed throughout the H9N2-infected spleen. In the GPV-infected spleen section, the GPV antigen was limitedly detected. Moreover, CD4- and CD8α-positive signs of the H9N2-infected group were more intense than those of the GPV-infected group (Figure 1D).

### 2.2. Transcriptional Data Profile of the Virus-Infected Goslings

The cDNA libraries of nine goose spleen samples were constructed for sequencing in an Illumina HiSeq™ 2000 machine (Illumina, CA, USA). Through Solexa RNA sequencing, vast raw data were obtained. After the low-quality reads were removed, at least 43 million 100-bp paired-end reads were generated for each sample. Moreover, the valid ratio of the reads was close to 95% (Table 1). The number of reads in our study met the requirement of high-quality eukaryotic transcriptome reconstruction, requiring more than ten million reads to identify new genes [11]. Q30 means that the accuracy rate of the sequence base was 99.9% and was regarded as a measurement of the quality of the sequence. More than 93% of reads from all of the samples possessed a 99.9% accuracy rate of the sequence base (Table 1). In addition, the sequencing raw data of control group, GPV- and H9N2-infected groups have been deposited into the Short Reads Archive (SRA) database under the accession numbers SRR5006761, SRR5006762, and SRR5006763, respectively.

### 2.3. Differential Expression Pattern after Infection

**Figure 2:**
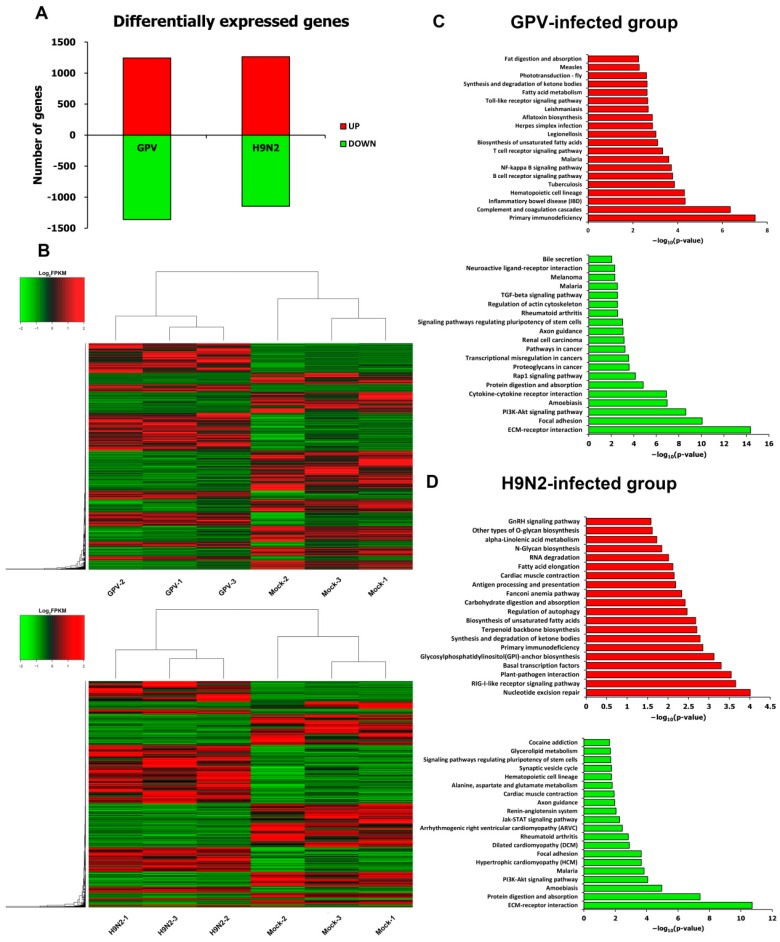
Analysis of the differentially expressed genes after infection with GPV and H9N2. (**A**) The number of differentially expressed genes after infection with GPV and H9N2; (**B**) hierarchical clustering analysis of differentially expressed genes in the GPV- and H9N2-infected groups; (**C**) KEGG enrichment analyses of the up- (red bar chart) and down-regulated genes (green bar chart) in the GPV-infected groups; and (**D**) KEGG enrichment analyses of the up-(red bar chart) and down-regulated genes (green bar chart) in the H9N2-infected groups. Only the top 20 pathways are listed here.

The threshold of a *p*-value ≤ 0.05 was used to identify the differentially expressed genes between the mock- and virus-infected groups. In total, 2604 unigenes showed a significant difference between the mock- and GPV-infected goose spleens, with 1243 up-regulated and 1361 down-regulated unigenes (Table S1). Compared with the mock-infected group, 2409 unigenes were significantly differentially expressed in the H9N2-infected group, with 1262 up-regulated and 1147 down-regulated unigenes (Table S2). Interestingly, the number of up-regulated unigenes were as high as the number of down-regulated unigenes in the two virus-infected groups (Figure 2A). However, within each virus-infected group, some of the up-regulated unigenes were distributed in diverse individuals, while almost all of the down-regulated genes were shared by different individuals. As shown in Figure 2B, GPV- or H9N2-infected individuals were different from mock-infected individuals and separately clustered into one group. Notably, most of the differentially expressed unigenes showed similar expression patterns among different individuals within each virus-infected group, indicating that the samples within different infected groups were connected closely to each other and had an important significance for further investigation.

To identify the pathways in which the differentially expressed unigenes were mainly involved, Kyoto Encyclopedia of Genes and Genomes (KEGG) enrichment analysis was conducted. Obviously, the up-regulated unigenes were mostly involved in the host-pathogen recognition and interaction. In the GPV-infected group, approximately three-quarters of the pathways among the top 20 pathways with enriched up-regulated unigenes were related to immunity, such as the B-cell receptor signaling pathway, NF-κB (NF-κB) signaling pathway, T-cell receptor signaling pathway, and Toll-like receptor signaling pathway (Figure 2C). In the H9N2-infected group, many up-regulated unigenes also enriched the pathways of the immune system, such as the RIG-I-like receptor signaling pathway, and antigen processing and presentation pathways. Strikingly, the extracellular matrix (ECM)-receptor interaction pathway was the top pathway enriched in down-regulated unigenes in the two virus-infected groups (Figure 2D).

### 2.4. Dynamics of Immune Relevant Genes during the Defense Response

**Figure 3:**
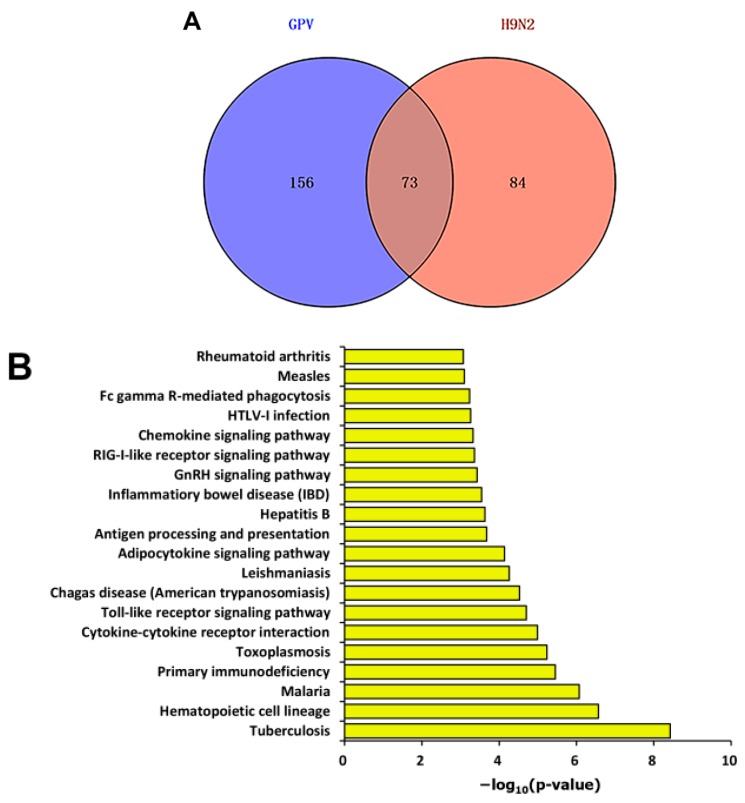
Co-immune relevant unigenes of the two virus-infected groups. (**A**) The Venn diagram shows the numbers of immune relevant unigenes that were either unique or shared between the GPV- and H9N2-infected groups; and (**B**) KEGG enrichment analysis of co-immune relevant unigenes of the two virus-infected groups.

Among the thousands of differentially expressed unigenes, 229 and 157 immune relevant genes were screened out from the GPV- and H9N2-infected groups, respectively (Tables S3 and S4). In total, 313 differentially expressed unigenes related to the immune response were identified in the virus-infected groups, among which 73 unigenes were shared between the two virus-infected groups. KEGG enrichment analysis was applied to the 73 co-immune relevant unigenes (Figure 3A). As shown in Figure 3B, the co-immune relevant unigenes were enriched not only in innate immune pathways, such as cytokine-cytokine receptor interaction and Toll-like receptor signaling pathways, but also in the adaptive immune pathways, including the antigen processing and presentation and Fc gamma R-mediated phagocytosis pathways.

**Figure 4:**
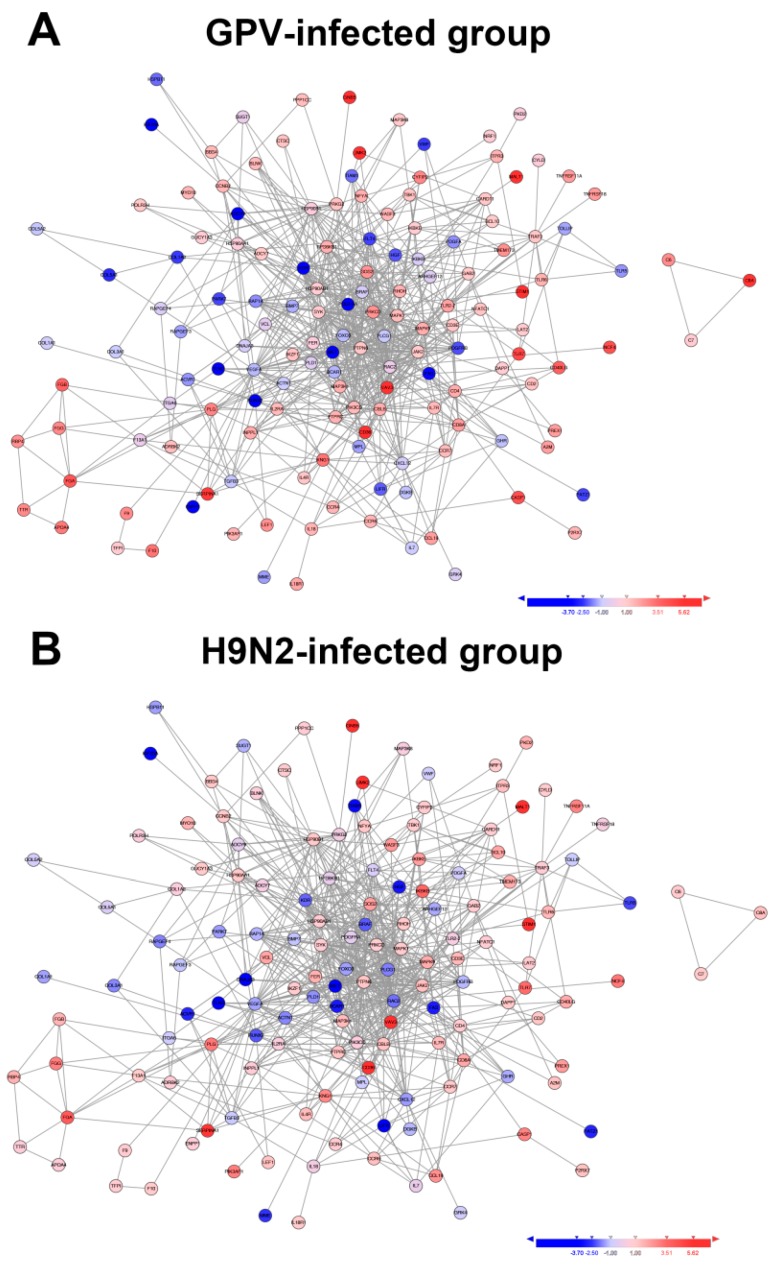
STRING analyses of co-immune relevant unigenes in the two virus-infected groups. All of the significant co-immune relevant unigenes were mapped to the database of the protein-protein interactions of chicken using STRING tool v9.05. (**A**) GPV-infected group; (**B**) H9N2-infected group. The expression fold change is shown as log_2_ (virus/mock) and is represented as indicated in the color scale.

To investigate the interaction of these proteins corresponding to the differentially expressed unigenes, 313 unigenes related to the immune response were analyzed using STRING v9.05 (Figure 4). Although the altered expression profiles of these unigenes between the GPV- and H9N2-infected groups were similar, the change in the magnitude of the genes in the GPV-infected group was greater than that of genes in the H9N2-infected group. Among the common up-regulated unigenes, guanine nucleotide-binding protein subunit β-5 (GNB5), guanine nucleotide exchange factor VAV3 (VAV3), and mucosa-associated lymphoid tissue lymphoma translocation protein 1 (MALT1) were largely increased. GNB5 and VAV3 participated in multiple signal transduction pathways [12,13]. MALT1 synergized with B-cell lymphoma 10 (BCL10) was identified to enhance the activation of NF-κB in lymphocytes [14]. Moreover, the two proteins played an essential role in the development of lymphocytes and the effector function of mature lymphocytes [15]. The interaction of differentially expressed immune-related genes indicated that immune molecules work not only independently but as a whole with mutual regulation to execute the antiviral function in the goose.

### 2.5. Innate Immune and Adaptive Immune Response after Infection with the Two Viruses

**Figure 5:**
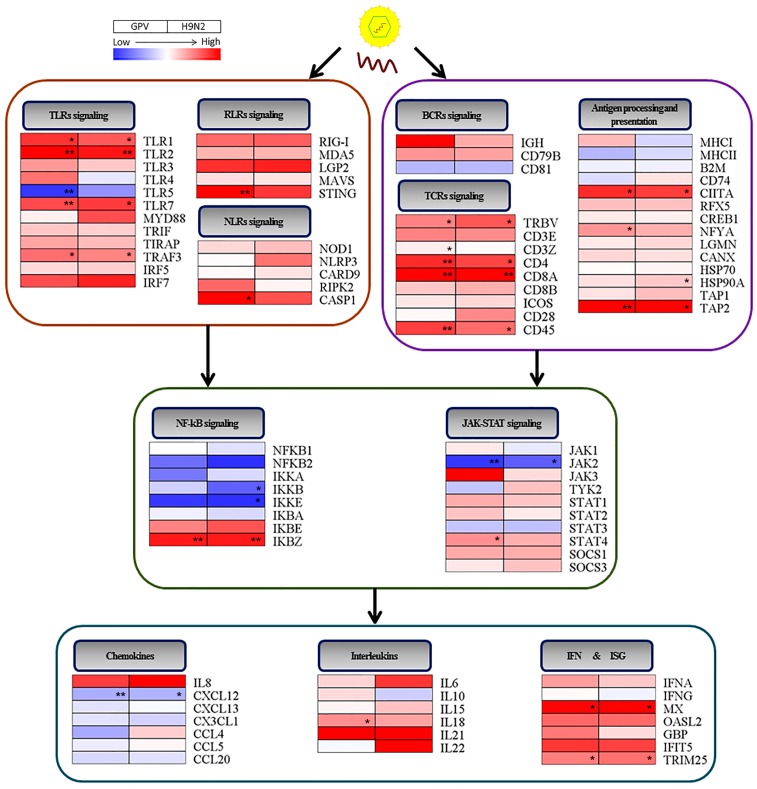
Comprehensive analysis of immune gene expression patterns after GPV and H9N2 infection. Four sections—PRR signaling pathways in the innate immune response, capture and display of the microbial antigen process in the adaptive immune response, cascade amplification signaling pathways, and cytokines/ISGs—were summarized. Among these pathways, genes that play pivotal roles in TLRs, RLRs, and NLRs signaling, as well as genes that encode interleukins, interferons, and ISGs were activated after viral infection. While, the genes associated with the activation of NF-κB signaling were depressed. The two grids in each line represent the GPV and H9N2 group, respectively. The colors in the grid represent the expression fold change as indicated in the color scale. The special fold-changes of these immune genes and *p*-value were listed in Table S5. * *p* ≤ 0.05, ** *p* ≤ 0.01.

Upon virus invasion, the host cell initiates a complex defense response mediated by the early phase of the innate immune reaction and the later adaptive immune reaction. In the innate immune system, the recognition of an invasive virus by the pattern-recognition receptors (PRRs) constitute an early line of defense [16]. Currently, four classes of PRRs have been identified, Toll-like receptors (TLRs), RIG-I-like receptors (RLRs), NOD-like receptors (NLRs), and C-type lectin receptors (CLRs). Reasonably, CLRs were not identified in our study because they were involved in fungal recognition, not virus recognition. Except for TLR4 in the H9N2-infected group and TLR5 in the two virus-infected groups, all members of the TLR family were up-regulated in GPV- and H9N2-infected goslings (Figure 5). In addition, the expression levels of TLR1, TLR2, TLR7, and TRAF3 were significantly increased in response to GPV and H9N2 infection. The RLR family is composed of the retinoic acid-inducible gene I (RIG-I; also known as DDX58), melanoma differentiation-associated gene 5 (MDA5; also known as IFIH1), and LGP2 (also known as DHX58) [17]. In our study, both the RIG-I and MDA5 genes were upregulated after GPV and H9N2 infection. Meanwhile, two adaptor molecules, mitochondrial antiviral signaling protein (MAVS; also known as IPS-1) and stimulator of interferon genes protein (STING; also known as TMEM173 and MITA), were activated in the GPV- and H9N2-infected gosling spleens, particularly, the expression level of STING was extremely increased in the GPV-infected group. Furthermore, the fold changes of LGP2 in the two virus-infected groups were both higher than 4.0. In the NLR signaling pathway, the expression levels of all five members were increased in the infected gosling spleens. Notably, CASP1 in GPV-infected gosling spleens was markedly up-regulated.

It is well accepted that complete clearance of intracellular viruses requires the destruction of infected cells by the adaptive immune system [18,19]. Antigen-presenting cells (APCs) capture microbial antigens and present them to T cells that initiate the adaptive immune response, which plays an essential role in the antigen processing and presentation pathway [20]. In the antigen processing and presentation group, most of the members were expressed at a higher level in both virus–infected groups than in the mock-infected group (Figure 5). The expression of major histocompatibility complex class I (MHC I), major histocompatibility complex class II (MHC II), β-2-microglobulin (B2M), and CD47 (also known as DHLAG, Ii) showed minor down-regulation in one of the two virus-infected groups or in both two groups. However, the expression levels of MHC class II transactivator (CIITA), regulatory factor X 5 (RFX5), CAMP responsive element binding protein 1 (CREB1), and nuclear transcription factor Y subunit A (NFYA), which are essential for the transcriptional activity of MHC I and MHC II, were increased following virus infection. Moreover, the expression of transporter associated with antigen processing 2 (TAP2) was also markedly increased in both virus-infected groups.

The second phase of the adaptive immune response is the activation of lymphocytes. APCs, such as dendritic cells (DCs), present antigens to naïve T lymphocytes to initiate the proliferation and differentiation of lymphocytes, subsequently eliminating the microbes by effector cells. Meanwhile, antigens can be directly bound to the surface receptors of B lymphocytes and trigger the B-cell receptor-mediated signaling pathway. In the T-cell receptor signaling pathway, the expression levels of all the molecules increased after GPV and H9N2 infection (Figure 5). Among these up-regulated unigenes, the T-cell receptor beta chain V region (TRBV), T-cell surface glycoprotein CD4 and activated T-cell surface glycoprotein CD8 α chain (CD8α) were significantly up-regulated in the virus-infected groups. However, the expression levels of the molecules that participated in the B-cell receptor signaling pathway slightly changed after infection with both viruses, without a significant difference between mock infection and virus infection.

The NF-κB pathway and Janus kinase/signal transducers and activators of transcription (JAK/STAT) pathway play central roles in directly translating an extracellular signal into the intra-nuclear transcription of a vast array of cytokines [21,22]. In this study, the expression patterns of genes involved in the nuclear NF-κB signaling pathway was demonstrated to be almost similar between the GPV- and H9N2-infected groups (Figure 5). Except NF-κB inhibitor ε (IκBε) and NF-κB inhibitor ζ (IκBζ), almost all members in NF-κB signaling pathway were down-regulated in two virus-infected goslings. Unlike the genes in the NF-κB pathway, most genes were increased in the JAK/STAT pathway after infection with both viruses. However, a significant down-regulation of Janus kinase 2 (JAK2) was observed in both the GPV- and H9N2-infected gosling spleens. In addition, suppressor of cytokine signaling 1 (SOCS1) and suppressor of cytokine signaling 3 (SOCS3) were increased in the two virus-infected groups.

Cytokines have become increasingly regarded as central regulators of the immune system [23] that are involved in virtually every aspect of immunity and inflammation, including innate immune cellular recruitment and activation, lymphocyte proliferation, differentiation and maturity, and antibody development [23]. In this study, the data showed that the change trend of cytokines after infection with GPV is consistent with that of the cytokine response to H9N2 infection (Figure 5). The enhancement of the expression levels of most interleukins were observed in both virus-infected groups, especially, the expression level of IL-18 in GPV-infected goslings. However, the expression levels of the chemokine decreased after infection with GPV and H9N2.

Interferons (IFNs) are well accepted as the most robust antiviral cytokines that respond against viral infection [24], but they do not execute their antiviral function directly. By binding to specific cognate receptors on the cell surface, IFNs will activate the JAK/STAT signaling pathway and induce the production of ISGs [25], which are the primary effectors of the IFN response [26]. The data indicated that goose IFNα was induced following the up-regulation of ISGs in response to infection with both viruses. Notably, the expression levels of goose myxovirus resistance protein (Mx) and tripartite motif containing 25 (TRIM25) were markedly increased in both virus-infected groups.

**Figure 6:**
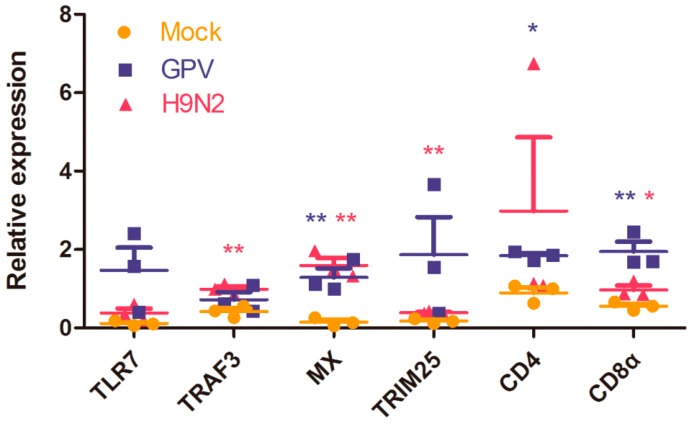
Expression profiles of six differentially expressed genes were verified by RT-qPCR. All of these genes play important roles in viral defense. The mRNA levels of these genes were increased after viral infection, a finding that was consistent with the altered expression profiles in the transcriptome data. Each dot represents a goose individual and different colors show different experimental groups (saffron yellow, blue, and pink represent mock-, GPV-, and H9N2-infected groups, respectively). Data are represented as the mean ± SEM. * *p* ≤ 0.05, ** *p* ≤ 0.01.

Finally, the expression levels of six differentially expressed genes (TLR7, TRAF3, Mx, TRIM25, CD4, and CD8α) implicated in virus defense were verified by real-time quantitative PCR (RT-qPCR) (Figure 6). The expression trend of genes evaluated by RT-qPCR was consistent with the results of transcriptome data (Figure 5 and Table S5).

## 3. Discussion

Upon virus invasion into the host, a complex and fierce battle is conducted between the host cells and viruses. With the continuous replication of the viruses in the host cells, the host organism becomes damaged to different degrees. In the present study, two pathogens seriously impact the growth of body weight, especially H9N2. In addition, the changes in the body weights of two virus-infected goslings displayed a similar variation trend: the body weight of goslings slowly increased until 4 dpi and simultaneously appeared to be lost at 5 dpi (Figure 1A). The two viral antigens survived in the virus-infected gosling spleens (Figure 1D), causing diffuse hemorrhage in the spleen samples from the two virus-infected gosling groups (Figure 1C). On the other hand, the down-regulated unigenes in the two virus-infected groups enriched in the “ECM-receptor interaction” pathway, indicating that the two viruses caused serious defects in the deposition of the ECM and expression of adhesion receptors on the surface of the host cells. Moreover, the defects in the ECM proteins and receptors will affect development, as has been confirmed in multiple organisms [27,28], explaining, to a certain extent, the body weight loss of goslings after infection with both viruses.

In the two infective groups, many genes that were up regulated directly or indirectly participate in the immune response pathway (Figure 2), including TLR7, TRAF3, CD4, and CD8α (Figure 5 and Figure 6). Moreover, CD4- and CD8α-positive cells were detected in the two virus-infected spleen groups, indicating that the immune system of the infected goslings was activated (Figure 1D). Since the epithelial cell line barrier was destroyed by invasive viruses, the innate and adaptive immune responses were initiated to defend against the viruses. Additionally, the initiation of the innate immune response is mediated by pathogen recognition through PRRs, including TLRs, RLRs, and NLRs. Different PAMPs with various structural components will react with specific PRRs [16]. Interestingly, infection with both viruses markedly promoted the expression of TLR1 and TLR2, which are sensors of bacterial components [29,30], which may be explained by the up-regulation of IFNα and IFNγ that could enhance the expression of TLR1, TLR2, TLR3, and TLR7 in viral infections [31]. Moreover, TLR7 can recognize viral single-stranded RNA (ssRNA) [32], the up-regulation of which might represent a positive feedback to the generation of ssRNA during the replication of GPV and H9N2. However, the expression level of TLR5 was decreased in both virus-infected groups, a result that was consistent with those found in H9N2-infected mice [33]. In the GPV-infected goslings, the host cells up-regulated TLR3, as well as RIG-I and MDA5—which have been identified to sense 5′-triphosphate ssRNA, secondary-structured RNA, and dsRNA in the cytoplasm [34]—to recognize dsRNA generated during GPV replication [35]. Moreover, the genome of GPV could be recognized by the dramatically activated STING, which is a cytosolic DNA sensor [36]. In addition, RIG-I plays an important role in evoking type I IFN responses to influenza virus infection [34]. Thus, it was no surprise that RIG-I was activated in H9N2-infected goslings. Furthermore, the increase in LGP2, as a negative regulator of RIG-I and MAD5 [37], indicated a mechanism of feedback control of the excessive inflammatory cytokines by the activation of RLRs signaling [38]. The activation of nucleotide binding and oligomerization domain 1 (NOD1) and pyrin domain-containing 3 (NLRP3), as intracellular sensors, was observed in both infected groups, leading to recruitment of the adaptor proteins, such as receptor-interacting serine-threonine kinase 2 (RIPK2; also known as RICK or RIP2) and caspase recruitment domain-containing protein 9 (CARD9) and subsequently resulting in the activation of downstream signaling pathways and the secretion of multiple cytokines [39]. Furthermore, both NOD1 and NLRP3 are essential for the formation of the caspase-1 (CASP1) inflammasomes. It has been reported that the activation of CASP1 can promote the maturation of IL-1β and IL-18 [18,40]. The significant activation of CASP1 in GPV-infected gosling spleens might be a possible explanation for the significant up-regulation of IL-18 in the GPV-infected group.

GPV and H9N2 were recognized by PRRs and captured by APCs, triggering the downstream signaling cascades and production of cytokines. IL-1β and IL-6, as the most important proinflammatory cytokines, were largely secreted in both virus-infected groups to induce inflammation and the recruitment of leukocytes. IFNα and IFNγ, two types of antiviral cytokines, were also up-regulated after viral infection, activating the JAK-STAT signaling pathways and inducing the transcription of ISGs, including Mx, OASL2, GBP, IFIT5, and TRIM25. In both virus-infected groups, these ISGs were up-regulated to different extents. Notably, Mx and TRIM25 were significantly up-regulated against viral infection. The former can prevent viral replication by trapping viral essential components and blocking the nuclear import of nucleocapsids [41] or interacting with viral ribonucleoprotein structures [42]; the latter participates in RIG-I-mediated innate immunity by inducing the ubiquitination of RIG-I, thereby stimulating the production of IFNs and eliciting the host antiviral response [43]. Strangely, most of the chemokines and genes involved in the NF-κB signaling pathway were widely depressed in both virus-infected groups. It is worth mentioning that IκBζ, a negative regulator of the NF-κB pathway, was markedly increased after infection with both viruses, a finding that was consistent with the downregulation of almost all of the genes involved in the activation of the NF-κB pathway. Moreover, SOCS1 and SOCS3, as suppressors of cytokine signaling, were activated, indicating that the infected goslings may avert organismic self-damage by the negative feedback regulation of the excessive immune response [44].

During the co-evolution of viruses with the host immune system, viruses possess various strategies to escape host immune detection. One of these strategies is to down-regulate the expression levels of genes involved in immune defense, such as MHC II [45]. It has been reported that the down-regulation of MHC II occurs in the lungs of H9N2-infected chickens [46], findings that are in accordance with the data in our study. Although no research has shown that the expression level of JAK2 is inhibited by GPV or H9N2, the varicella-zoster virus indeed inhibits the expression of JAK2 [47]. Inhibition of cellular apoptosis is another strategy to counter host immunity by viruses. Upon viral infection, the host cell evokes the NF-κB mediated apoptotic pathway to limit the replication and spread of the virus. Generally, NF-κB proteins are inactivated by a family of κB inhibitors (IκBs) in the cytoplasm. Upon inhibitor of nuclear factor κB kinases (IKKs) complex activation, the IκBs are phosphorylated and degraded, and then NF-κB dimers are released into the nucleus for the transcription of specific target genes, leading to cellular apoptosis and the induction of inflammatory cytokines [48]. In the present study, the decrease in IKKs (IKKα, IKKβ, and IKKε) and NF-κB proteins (NF-κB1 and NF-κB2), as well as the increase in IκBs (IκBε and IκBζ), indicate an insufficient amount of activated NF-κB to induce the expression of genes implicated in the apoptotic pathway. The depressed apoptotic process contributed to cell survival and viral replication. In addition, the expression levels of inhibitor of nuclear factor κB kinase subunit β (IKKβ) and inhibitor of nuclear factor κ-B kinase subunit epsilon (IKKε) were significantly inhibited by the H9N2 virus, a finding possibly explained by the ability of non-structural protein 1 (NS1) of influenza A to inhibit NF-κB activation [49]. Moreover, HSP70, an apoptosis inhibitor, that was also up-regulated in both virus-infected groups (Figure 5) can block the apoptotic process by interfering with caspase activation and downstream cytochrome c release [50], which is beneficial for viruses to escape host immune.

## 4. Materials and Methods

### 4.1. Ethics Statement

The animal studies were conducted in strict accordance with the National Institutes of Health guidelines for the performance of animal experiments. All of the animal experimental protocols were approved by the Institutional Animal Care and Use Committee of Sichuan Agriculture University, Chengdu, China (Protocol Permit Number: XF2014-18).

### 4.2. Animal Experiment and Spleen Tissue Collection

Twenty-seven one-day-old geese were purchased from the waterfowl breeding center of Sichuan Agriculture University and were randomly divided into three groups with nine geese in each group. One group of geese was infected with 500 µL of GPV (measured at 5 × 10^−6.6^ EID_50_/mL); the second group was infected with 500 µL of H9N2 (measured at 7 × 10^12.64^ copies/mL); and the last group was inoculated with an equivalent amount of normal saline. On 5 dpi, three geese from each group were euthanized, and their spleen tissues were collected immediately. One portion of each spleen tissue was cut into pieces and kept in RNAstore (CWBIO, Beijing, China) for RNA-seq. The second portion of samples was stored separately in liquid nitrogen for RT-qPCR analysis. Moreover, the remaining samples were subjected to tissue slice preparation for hematoxylin and eosin (H and E) and immunohistochemical (IHC) staining.

### 4.3. H & E Staining and Immunohistochemical (IHC) Analysis

The spleen samples were fixed in buffered formalin, dehydrated in graded alcohol, embedded in paraffin wax and cut into 5-µm thick sections. Some sections were stained with H & E as the routine histopathological staging. Concurrently, immunohistochemical reactions were performed according to a previous description with slight modifications [51]. One part of each section from H9N2-infected spleens and GPV-infected spleens was incubated with rabbit polyclonal anti-H9N2 HA antibody (Sino Biological Inc., Beijing, China) and mouse polyclonal anti-GPV antibody (provided by our laboratory), respectively. The remaining sections were incubated with mouse anti-duck CD4 monoclonal antibody (AbD Serotec, Kidlington, UK) or mouse anti-duck CD8a monoclonal antibody (AbD Serotec, Kidlington, UK). Biotinylated goat anti-rabbit or goat anti-mouse (ZSGB-BIO, Beijing, China) immunoglobulin G was incubated as the secondary antibody.

### 4.4. Total RNA Isolation and Illumina Sequencing

The stored spleen tissues were lysed, and total RNA was extracted using TRIzol reagent (Invitrogen, Carlsbad, CA, USA) according to the manufacturer’s protocol. For transcriptome analysis, the concentration and quality of the RNA were assessed using the NanoDrop system (Thermo Fisher, Waltham, MA, USA) and Agilent 2100 Bioanalyzer (Agilent Technologies, Santa Clara, CA, USA). The RNA integrity numbers (RINs) of samples were obtained to measure the quality of RNA in a standard manner [52]. The cDNA libraries were generated using the TruSeq RNA Sample Preparation Kit v2 (Ilumina, San Diego, CA, USA) and then were loaded onto the Illumina HiSeq™ 2000 Instrument for sequencing. Finally, the raw reads generated from the sequencing run were evaluated using FASTQC and NGS QC TOOLKIT (Section 2.3) [53], and then the clean reads from nine samples were de novo spliced into unigenes using Trinity software (vesion:trinityrnaseq_r20131110) [54] and TGICL [55].

### 4.5. Transcriptome Data Analysis

The annotation of the goose reference genome was unreleased when we analyzed the data so that all of the assembled unigenes were annotated as previously described [56]. The expression level of each unigene in the samples were calculated and normalized by fragments per kilobase of unigene per million mapped fragments (FPKM) [57], and the differentially expressed genes were screened based on a *p*-value that was adjusted by the false discovery rate (FDR). The threshold for the differentially expressed genes was a *p*-value ≤ 0.05. Subsequently, hierarchical clustering of the differentially expressed genes was performed using GeneSpring software (Agilent Technologies, Santa Clara, CA, USA, v11.0) to visualize the differential gene expression patterns and correlation among the samples. Finally, Gene Ontology (GO) and KEGG pathway enrichment analyses were conducted to determine the pathways in which the differentially expressed unigenes mainly participated.

### 4.6. RT-qPCR Analysis

Total RNA was isolated separately using TRIzol reagent (Invitrogen, Carlsbad, CA, USA) following the manufacturer’s protocol and then was reverse transcribed into cDNA using 5× All-in-One RT MasterMix (Abm, Milton, ON, Canada). Quantitative analysis of candidate gene from each sample was performed using the EvaGreen 2× qPCR MasterMix Kit (Abm, Milton, ON, Canada) with the Bio-Rad CFX96 Real-Time Detection System (Bio-Rad, Hercules, CA, USA). The optimal primers were synthesized by Beijing Genomics Institute (Beijing, China), and the sequences are listed in Table 2. The relative gene expression of each gene was calculated using the Livak and Schmittgen 2^−ΔΔ*C*t^ method [58] and was normalized to goose *GAPDH*. The differential mRNA expression levels of the candidate genes in each sample were verified in triplicate.

### 4.7. Bioinformatics Analysis

The Venn diagram that presented the numbers of immune-related differentiall- expressed unigenes that were either unique or shared between the two infection groups was constructed using the online software Venny (Available online: http://bioinfogp.cnb.csic.es/tools/venny/). The RT-qPCR data were analyzed with Bio-Rad CFX Manager Software and GraphPad Prism 5. Significantly and differentially expressed unigenes related to the immune response were mapped to the database of chicken protein-protein interactions in STRING tool v9.05, and the visualization networks of proteins were composited by cytoscape v3.0.2 (Available online: www.cytoscape.org/).

## 5. Conclusions

The data provide a comprehensive transcriptome profile of the GPV- and H9N2-infected gosling spleen tissues. Although GPV and H9N2 possess diverse nucleic acid genomes, the immune relevant genes in both virus-infected groups displayed similar expression patterns (Figure 5), with almost identical fold change in important immune-related genes (Table S5) which may be explained by common components that are shared by viruses and similar viral nucleic acids are produced during replication. Moreover, the up-regulation of immune-related genes and the depression of NF-κB signaling may facilitate the elucidation of the molecular mechanisms of the host-pathogen interaction.

## Figures and Tables

**Table 1:** Statistics of the RNA-seq datasets.

Sample	RINs	Raw Reads	Clean Reads	Q30 (%)	GC Content (%)
Mock-1	9.9	55,922,366	55,647,608	94.02	47.00%
Mock-2	9.9	45,717,764	45,490,282	93.94	47.50%
Mock-3	10.0	49,752,976	49,480,934	94.09	47.00%
GPV-1	10.0	54,915,460	54,607,164	93.77	47.00%
GPV-2	9.9	50,402,808	50,120,838	93.78	47.00%
GPV-3	9.9	43,356,716	43,137,380	94.03	47.00%
H9N2-1	9.9	49,921,838	49,641,084	94.03	46.00%
H9N2-2	9.8	50,382,454	50,133,860	94.17	47.00%
H9N2-3	9.9	54,094,468	53,823,010	94.00	47.50%

**Table 2:** Primers used for RT-qPCR analysis.

Gene Symbol	Forward Primer (5′–3′)	Reverse Primer (5′–3′)	Size/bp
*IL-1β*	TCCGCCAGCCGCAAAGTG	CGCTCATCACGCAGGACA	136
*IL-6*	GCTTTGTGAGGAGGGATT	CCGTTAGACACTGGGGTT	120
*CD8α*	AGAGACGAGCAAGGAGAA	GACCAGGGCAATGAGAAG	97
*CD4*	TTTCAACGCCACAGCAGA	GTGCCTCAACTGGATTTT	127
*Mx*	TTCACAGCAATGGAAAGGGA	ATTAGTGTCGGGTCTGGGA	183
*TRIM25*	CCACCACCCTCAGCGTTTC	GCCATAGCAGATGCCAAT	127
*TAP2*	TCTTCCAGCAGACCACAGC	AAGGGGCACCTCAAGCAG	188
*TLR7*	CACAGAAAAATGGTACCTC	TACATCGCAGGGTAAACT	117
*GAPDH*	CATCTTCCAGGAGCGCGACC	AGACACCGGTGGACTCCACA	80

## Supplementary Materials

Supplementary materials can be found at www.mdpi.com/1422-0067/17/12/1990/s1.
